# Interplay between Endothelin and Erythropoietin in Astroglia: The Role in Protection against Hypoxia

**DOI:** 10.3390/ijms15022858

**Published:** 2014-02-19

**Authors:** Richard Schäfer, Lars Mueller, Reinhild Buecheler, Barbara Proksch, Matthias Schwab, Christoph H. Gleiter, Lusine Danielyan

**Affiliations:** 1 Institute for Transfusion Medicine and Immunohaematology, German Red Cross Blood Donor Service Baden-Württemberg-Hesse gGmbH, Johann-Wolfgang-Goethe-University Hospital, Sandhofstrasse 1, Frankfurt/Main D-60528, Germany; E-Mail: r.schaefer@blutspende.de; 2 Institute for Clinical and Experimental Transfusion Medicine (IKET), University Hospital of Tuebingen, Otfried-Müller-Strasse 4/1, Tuebingen D-72076, Germany; 3 Department of Clinical Pharmacology, Institute of Clinical and Experimental Pharmacology and Toxicology, University Hospital of Tuebingen, Auf der Morgenstelle 8, Tuebingen D-72076, Germany; E-Mails: drlarsmueller@freenet.de (L.M.); pharmakologie@gmx.net (R.B.); barbara.proksch@uni-tuebingen.de (B.P.); matthias.schwab@ikp-stuttgart.de (M.S.); christoph.gleiter@med.uni-tuebingen.de (C.H.G.); 4 Dr. Margarete-Fischer-Bosch Institute of Clinical Pharmacology, Auerbachstrasse 112, Stuttgart D-70375, Germany

**Keywords:** endothelin, erythropoietin, astroglia, neuroprotection, hypoxia, endothelin receptor type A antagonist, aging

## Abstract

We show that, under *in vitro* conditions, the vulnerability of astroglia to hypoxia is reflected by alterations in endothelin (ET)-1 release and capacity of erythropoietin (EPO) to regulate ET-1 levels. Exposure of cells to 24 h hypoxia did not induce changes in ET-1 release, while 48–72 h hypoxia resulted in increase of ET-1 release from astrocytes that could be abolished by EPO. The endothelin receptor type A (ETA) antagonist BQ123 increased extracellular levels of ET-1 in human fetal astroglial cell line (SV-FHAS). The survival and proliferation of rat primary astrocytes, neural precursors, and neurons upon hypoxic conditions were increased upon administration of BQ123. Hypoxic injury and aging affected the interaction between the EPO and ET systems. Under hypoxia EPO decreased ET-1 release from astrocytes, while ETA receptor blockade enhanced the expression of EPO mRNA and EPO receptor in culture-aged rat astroglia. The blockade of ETA receptor can increase the availability of ET-1 to the ETB receptor and can potentiate the neuroprotective effects of EPO. Thus, the new therapeutic use of combined administration of EPO and ETA receptor antagonists during hypoxia-associated neurodegenerative disorders of the central nervous system (CNS) can be suggested.

## 1. Introduction

Endothelin (ET), a potent vasoconstrictor peptide (*M*_W_: 2.492 kDa), was isolated for the first time in 1988 from porcine endothelial cells (ECs) [1]. Among the three isoforms (ET-1, ET-2, and ET-3), ET-1 is the most abundant and physiologically relevant isoform. ET-1 was shown to be expressed in ECs and also in tissues such as liver, lung, kidney, and brain [2,3]. The expression of the *ET* gene is increased by adrenalin, vasopressin, angiotensin II, growth factors, and cytokines, whereas nitric oxide, natriuretic peptide and heparin decrease the *ET* gene expression [4]. ETA and ETB receptors feature different binding affinities for ET isoforms and differ from each other by their amino acid sequences. The ETA receptor is known as a high affinity receptor for ET-1 whereas the ETB receptor shows an equal affinity for all ET isoforms [5]. Functionally, the ETA receptor mediates the classical vasoconstricting action via G protein activation, whereas the ETB receptor comes in two subtypes: activation of ETB1 receptor results in vasodilatation while ETB2 receptor mediates vasoconstriction [6,7].

In addition to its striking effect on neuronal survival [8], ET-1 also regulates numerous astroglial functions such as control of ion channel activity, glutamate uptake and secretion, glucose utilization, gap junction permeability, and calcium signaling [9–15]. Astrocytes can produce ET-1 and contribute to the homeostasis of ET-1 in the central nervous system (CNS) [16,17]. Astrocytes express both ETA and ETB receptor subtypes [17]. ET-1 induces cortical spreading depression via activation of the ETA receptor/phospholipase C pathway *in vivo*, and application of ET-1 can promote exacerbation of ischemic damage of the brain [18–20]. Blockade of ETA receptor limits acute neuronal posttraumatic injury in the cortex and the deficiency of ETB receptor was associated with an increased rate of neuronal apoptosis in the dentate gyrus [21,22]. Notably, ET-1-mediated dopaminergic dysfunction could be inhibited by selective ETB receptor blockade but not by BQ123, a selective ETA receptor antagonist [23]. Moreover, ETB receptor agonists were recently shown to exhibit a protective effect against neurotoxicity of amyloid beta (Aβ), a major contributor in the pathogenesis of Alzheimer’s disease [24].

The role of erythropoietin (EPO) in neuroprotection and neuroregeneration has been a subject of a various experimental and clinical studies in the past. EPO is expressed in hematopoietic and non-hematopoietic cell types, such as neurons, astrocytes, and renal interstitial fibroblasts [25]. EPO is considered a potent neuroprotective agent inducing anti-apoptotic and proliferative effects on cells in the CNS after hypoxic or traumatic injury, during metabolic stress, HIV-induced apoptosis, multiple sclerosis (MS), and Aβ induced neuronal apoptosis [26–29]. Moreover, EPO plays a crucial role in neuroprotection of stem cells. In detail EPO abolishes hypoxia-induced death of neuronal progenitors *in vitro* [30], acts synergistically with mesenchymal stem cells (MSCs) transplants to potentiate postischemic neurogenesis in a rat model of cerebral ischemia, and protects MSCs from hypoxia and glutamate-induced damage [31,32].

An increasing number of findings point toward similarities between protective mechanisms and possible interactions of ET and EPO signalling systems in the brain and other organs [30,33]. EPO increases the systemic and local tissue levels of ET-1 in peripheral organs such as kidney, liver and heart [34,35]. Hypoxia increases systemic as well as local EPO- and ET-1 production in different organs [36,37]. Both, EPO and ET, are linked to JAK/STAT and MAPK intracellular signalling [38–41]. In the CNS, both, EPO and ET, play pivotal roles in repair after tissue damage or cellular stress. EPO enhances neurogenesis and provides neuroprotection after stroke [42]. Its anti-inflammatory effect on the CNS was shown in a model of experimental autoimmune encephalomyelitis and there is evidence that EPO may improve motor function in MS patients [43,44]. Moreover, EPO has been shown to protect dopaminergic neurons and to improve neurobehavioral outcome in a rat model of Parkinson’s disease [45]. While neuroprotective effects of EPO after brain injury are unambiguous, ET-1 can contribute either to exacerbation of ischemic damage [19,46] or to improved survival of astrocytes during hypoxic/ischemic injury [47]. Thus, the neuroprotective effects of ET-1 might be mediated mainly by the ETB receptor [33].

To date, details of the crosstalk between ET and EPO systems and mechanisms of this interaction in the CNS remain unexplored. Both systems are important for organ-specific cellular defense during pathological conditions of the CNS and understanding their complex interactions could contribute to the development of novel therapeutic agents to promote neuroprotection.

Hence, in this study we investigated whether: (1) ET receptor blockade influences the production of EPO in astroglia; (2) EPO affects the production/release of ET-1 in human astrocytes; and (3) the ETA receptor blockade regulates the expression of EPO receptor (EPOR) in the CNS-cells.

## 2. Results

### 2.1. Influence of ETA Receptor Blockade on the Expression of EPO mRNA

**Figure 1.**
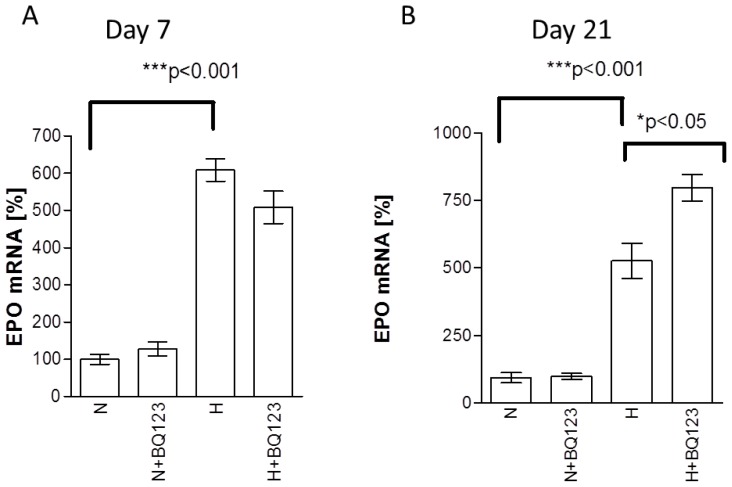
Expression of *EPO* mRNA in DIV7 (**A**) and DIV21 (**B**) neonatal rat astroglial primary culture treated with BQ123 upon normoxic (N) and hypoxic (H) conditions. The data are shown in percentage to normoxic control (N).

To reveal the age dependent changes of EPO expression in astroglial cells upon hypoxia and exposure to BQ123 we used short term (7 days *in vitro* [DIV7]) and culture-aged (21 days *in vitro* [DIV21]) neonatal rat astroglial primary culture. Treatment of DIV7 astrocytes with BQ123 under normoxic conditions did not reveal significant changes in EPO mRNA expression compared to untreated normoxic controls (Figure 1A, N *vs*. N + BQ123). Hypoxia induced 5-fold increase of EPO mRNA (*** *p* < 0.001, 508% ± 29% difference, Figure 1A, N *vs*. H). However, BQ123 treatment under hypoxic conditions did not significantly change the expression of EPO mRNA in comparison with hypoxic untreated control (Figure 1A, H + BQ123 *vs*. H).

Treatment of DIV21 astrocytes with BQ123 under normoxic conditions did not reveal any changes in *EPO* mRNA expression compared to untreated normoxic controls (Figure 1B, N *vs*. N + BQ123). Similar to DIV7 astrocytes 21-day old astrocytes responded to hypoxia with a five-fold increase in *EPO* mRNA (432% ± 67%, *** *p* < 0.001, Figure 1B, N *vs*. H). However, in contrast to the DIV7 astrocytes 21-day old cells responded to BQ123 with additional increase of *EPO* mRNA expression upon hypoxia (* *p* < 0.02, 271% ± 86% increase, Figure 1B, H *vs*. H + BQ123).

### 2.2. ETA Receptor Blockade Increases the Release of ET-1 Protein from Human Astrocytes

We hypothesized that the observed increase of *EPO* mRNA expression by ETA receptor blockade during hypoxia could be partially mediated by ET-1 (via ETB receptor). Therefore, we assessed the concentration of ET-1 protein released from human fetal astroglial cell line (SV-FHAS) under normoxic and hypoxic conditions.

**Figure 2.**
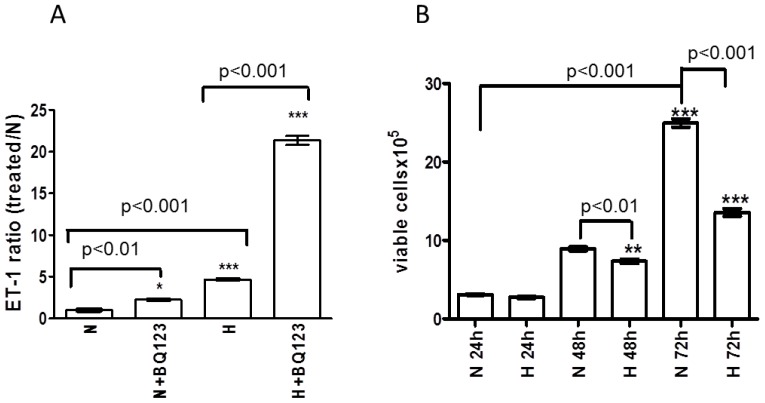
Quantification of viable human astrocytes and their ET-1 release into the culture medium. (**A**) ET-1 release from human astrocytes was measured 48 h after incubation with BQ123 under normoxic (N) and hypoxic (H) culture conditions. The data are presented as ratio of ET-1 concentration in treated cells to the mean of normoxic control (N); (**B**) Quantification of viable cells in human astrocytes culture. Trypan blue-negative viable cells were counted after 24, 48 and 72 h exposure to hypoxia (H 24 h, H 48 h, H 72 h) and compared with the respective normoxic controls (N 24 h, N 48 h, N 72 h).

Hypoxia induced 3.6-fold increase in ET-1 release (increased ET-1 release ratio) from astrocytes compared to normoxic control (Figure 2A, H *vs*. N, *p* < 0.001). BQ123 treatment slightly increased the ET-1 concentration in the cell culture supernatant under normoxic conditions (Figure 2A, N + BQ123 *vs*. N, *p* < 0.05), whereas, a strong (16-fold) increase in ET-1 release from BQ123-treated astrocytes was observed upon hypoxia (Figure 2A, H + BQ123 *vs*. H, *p* < 0.001).

### 2.3. Influence of Prolonged Hypoxia on the Viability of Human Astrocytes

The ET-1 release from SV-FHAS shown in Figure 2A was normalized to 1 × 10^6^ cells. However, the density of astrocytes in culture may affect the production and release of secreted factors, such as ET-1. Further, we addressed the question whether the impaired ability of astrocytes to produce and secrete ET-1 after 72 h exposure to hypoxia might be affected by the number of viable cells. Hence, the numbers of viable astrocytes under normoxic and hypoxic conditions were assessed at different time points (24, 48, and 72 h).

Exposure to hypoxia for 24 h did not affect the viability of astrocytes compared to normoxic control (Figure 2B, H 24 h *vs*. N 24 h). At time point 48 h the number of cells under normoxic conditions was significantly increased compared to normoxic samples from the time point 24 h (2.8-fold increase *p* < 0.001, Figure 2B, N 24 h *vs*. N 48 h). Hypoxia at time point 48 h slightly decreased the number of viable cells (Figure 2B, H 48 h *vs*. N 24 h), whereas, a nearly two-fold decrease in cell number was observed after 72 h exposure to hypoxia compared to normoxic control (Figure 2B, H 72 h *vs*. N 72 h). The fact that 48 h hypoxia decreased rather than increased the number of viable SV-FHAS confirms its direct influence on ET-1 release from astrocytes without involvement of an indirect effect through increased cell proliferation.

Under normoxic conditions after 72 h, an eight-fold increase of astrocyte proliferation was observed in contrast to the time point 24 h (Figure 2B, N 72 h *vs*. N 24 h).

### 2.4. Influence of EPO on the Release of ET-1 Protein from Human Astrocytes

Next, we addressed possible regulatory effects of EPO on the ET system in human astrocytes. The release of ET-1 protein into astrocyte culture supernatant was quantified by ELISA after exposure to hypoxia for 24, 48, and 72 h, with and without EPO (5 U/mL).

**Figure 3.**
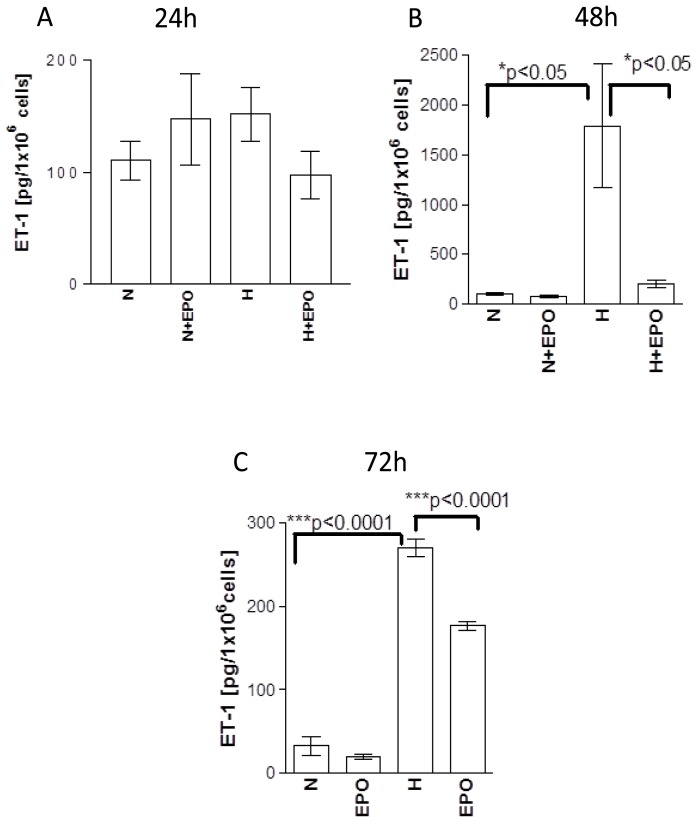
Release of ET-1 from human astrocytes (**A**) 24 h; (**B**) 48 h; and (**C**) 72 h after incubation with EPO under normoxic (N) and hypoxic (H) culture conditions.

At the first time point (24 h) no difference of ET-1 release could be observed between the groups. Neither hypoxia nor application of EPO under normoxic (N in Figure 3A) or hypoxic (H in Figure 3A) conditions showed an influence on the release of ET-1 from astrocytes (Figure 3A, N *vs*. N + EPO, H and H + EPO). However, application of EPO showed a trend to decrease ET-1 release upon hypoxia compared to hypoxic control (Figure 3A, H *vs*. H + EPO).

The basal release of ET-1 under normoxic conditions was similar between the groups after 24, 48 and 72 h (*cf.* N in Figure 3A–C). In contrast to the 24 h time point ET-1 release after 48 h incubation under hypoxic conditions showed a 10-fold increase compared to normoxia (Figure 3A, N *vs*. H). EPO treatment of hypoxic samples resulted in an eight-fold decrease of ET-1 release in comparison with hypoxic control (Figure 3A, *cf.* H with H + EPO) whereas no changes of ET-1 release could be observed after application of EPO under normoxic conditions (Figure 3A, N *vs*. N + EPO).

Under hypoxic conditions at time point 72 h astrocytes released 270.8 ± 10.09 pg ET-1/10^6^ cells, whereas 1789 ± 624 pg ET-1/10^6^ cells were released from astrocytes at time point 48 h under hypoxic conditions (*cf.* Figure 3B, H with Figure 3C, H). Similar to the results from time point 48 h, EPO treatment decreased the release of ET-1 upon hypoxia after 72 h incubation (Figure 3C, H *vs*. H + EPO).

### 2.5. Effects of ETA Receptor Blockade on the Expression of EPO Receptor in Astrocytes and Neural Precursors

In order to test whether expression of EPO receptor (EPOR) can be regulated by the ET system in CNS cells double immunostaining of EPOR and glial fibrillary acidic protein (GFAP) as a marker of astrocytes was performed in DIV7 and DIV21 rat astroglial primary cultures upon normoxic and hypoxic conditions.

**Figure 4.**
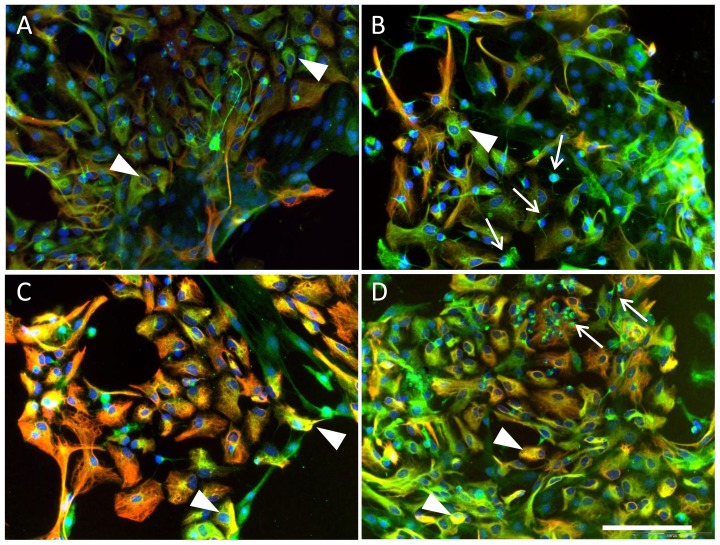
Expression of EPOR (green fluorescence) and GFAP (red fluorescence) in DIV7 neonatal rat astroglial primary culture. Cell nuclei are stained with DAPI (blue). (**A**) Normoxic control; (**B**) Cultures treated with BQ123 during normoxic conditions; (**C**) Hypoxic control; and (**D**) Cells treated with BQ123 during hypoxia. Arrows indicate the EPOR-positive bona fide neural precursors negative for GFAP; Arrowheads demonstrate the EPOR/GFAP-positive astrocytes. Scale bar in **A**–**D** 100 μm.

Analysis of short-term (DIV7) cultures showed an increase in intensity of EPOR expression in astrocytes (EPOR/GFAP positive cells in Figure 4) treated with BQ123 under normoxic conditions (Figure 4, arrowhead in B) compared to the normoxic control (Figure 4, arrowhead in A). The appearance of small round cells positive only for EPOR was observed in cultures exposed to BQ123 upon normoxic conditions (Figure 4, arrows in B), whereas, the normoxic control cultures contained mainly cells positive for EPOR and GFAP (Figure 4A).

An intense staining of EPOR in GFAP-positive astrocytes was observed under hypoxic conditions in cells (Figure 4, arrowheads in C). After application of BQ123 under hypoxia small EPOR-positive bona fide precursor cells negative for GFAP could be observed (Figure 4, arrows in D), and the majority of astrocytes expressed EPOR (Figure 4, arrowheads in D). The density of cells under normoxic (Figure 4A) and hypoxic (Figure 4C) control conditions was similar, whereas, the application of BQ123 under hypoxic conditions resulted in higher density of both GFAP-positive and negative cells (Figure 4D) hinting at the proliferative effect of ETA blockade under hypoxic conditions.

**Figure 5.**
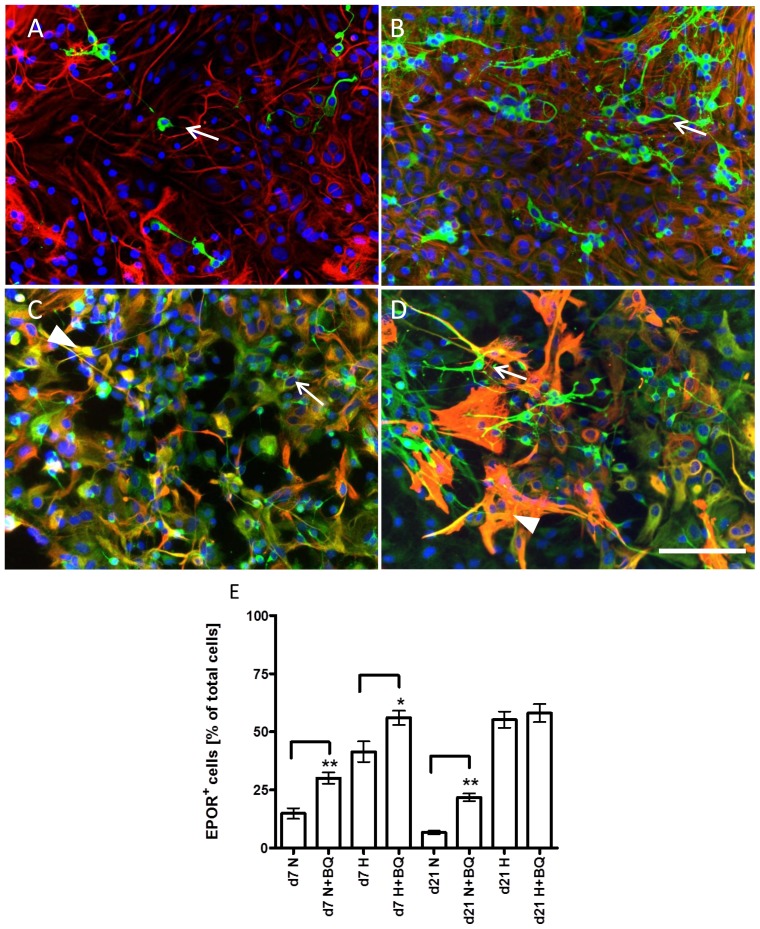
Expression of EPOR (green fluorescence) and GFAP (red fluorescence) in rat astroglial primary culture. Cell nuclei are stained with DAPI (blue). (**A**) Normoxic control (DIV21); (**B**) Cultures treated with BQ123 during normoxic conditions (DIV21); (**C**) Hypoxic control (DIV21); (**D**) Cells treated with BQ123 during hypoxia (DIV21). Arrows indicate the EPOR-positive bona fide neural precursors and neurons negative for GFAP. Arrowheads demonstrate either EPOR−/GFAP+ astrocytes (**A**,**B**) or those positive for both EPOR and GFAP (**C**,**D**). Scale bar in **A**–**D** 100 μm ; and (**E**) Quantification of EPOR-positive cells in astroglial primary cultures (DIV7 and DIV21) normalized to the mean of the total amount of cells per image (*n* = 3) counted by DAPI. *p* < 0.05 was considered as significant (* *p* < 0.05, ** *p* < 0.01).

Long term astroglial primary cultures (DIV21) under normoxic untreated control conditions contained mainly differentiated astrocytes as seen by the strong GFAP staining and phenotype. The intense EPOR staining was seen exclusively in bona fide precursor cells with round shape with or without short processes (Figure 5, arrows in A). The normoxic untreated control cell culture contained only few EPOR-positive *bona fide* precursor cells (Figure 5A), whereas the application of BQ123 under normoxic conditions increased the number of these cells and induced a neuron-like phenotype. Most of the EPOR-positive cells showed the long processes and small round bodies characteristic for neurons (Figure 5, arrows in B).

Under hypoxic control conditions the prominent staining of EPOR appeared, not only in immature precursor cells negative for GFAP (Figure 5, arrow in C), but also in astrocytes (Figure 5C, arrowhead). Similar to normoxic condition, application of BQ123 upon hypoxia led to appearance of neuron-like differentiated EPOR positive cells (Figure 5, arrows in D), while EPOR in astrocytes remained strongly expressed (Figure 5, arrowhead in D). Quantification of EPOR-positive cells in short (DIV7) and long-term cultures (DIV21) showed a significant increase in number of EPOR-positive cells induced by BQ123 in normoxic cultures (Figure 5E). Both, DIV7 and DIV 21, cultures responded to hypoxia exposure with a prominent increase in number of EPOR-positive cells. While the short-term cultures (DIV7) exposed to hypoxia responded to BQ123 administration with additional increase in number of EPOR-positive cells, no changes were seen in long term cultures (DIV21) after BQ123 administration (Figure 5E).

## 3. Discussion

In our study we observed the influence of BQ123 on the expression of *EPO* mRNA in “aged” astroglia only under hypoxia but not during normoxic conditions hinting at the peculiarity of astroglial response during hypoxia. We hypothesize that the increase of *EPO* mRNA expression after treatment with BQ123 could be mediated by two mechanisms: (1) hypoxia might increase the production and release of ET-1 that subsequently cannot be utilized through the ETA receptor due to blockade by BQ123; and/or (2) ET-1 production and release could be increased by its direct (via ETB receptor) or indirect (activation of different signaling pathways, inflammatory cytokines) actions on the expression of *EPO* mRNA. It was shown previously that selective ETB receptor stimulation can increase the release of inflammatory cytokines such as interleukin (IL)-6 or tumor necrosis factor (TNF)-α in rat astrocytes treated with lipopolysaccharide [48]. ET-1 acting through its ETB receptor may indirectly increase the expression of *EPO* mRNA by induction of TNF-alpha release. This cytokine was shown to increase the production of HIF1-α [49] that in turn can induce the expression of *EPO* mRNA [50].

We detected a prominent increase of ET-1 protein release from human astrocytes exposed to hypoxia. TNF-alpha can increase the release of ET-1 during hypoxia in astrocytes [51]. Therefore, a possible mechanism of increased ET-1 release from astrocytes during hypoxia might be the increased TNF-alpha release from these cells during hypoxic conditions [52]. We detected an increase of ET-1 release from human astrocytes after BQ123 treatment, and we propose two potential mechanisms that might act synergistically. (1) The blockade of ETA receptor could increase the availability of ET-1 to ETB receptor which consequently might trigger the production of ET-1 via ETB receptor [53] and/or (2) Due to the availability of ET-1 to ETB receptor through ETA blockade, ET-1 could activate the Protein Kinase-C via ETB receptor which in turn might result in increased expression of ET-converting enzyme-1 [54] converting the “premature” ET peptide into the “mature” ET peptide [55].

To date, storage and secretion of ET in neurons and glial cell is poorly understood. In cultured ECs, ET is not stored in vesicle structures and secretory granules [56]. Therefore, it is hypothesized that ET protein might be synthesized and secreted by a constitutive pathway on demand, since the main regulatory mechanism for ET-1 protein production occurs at the transcriptional level [56]. It cannot be excluded that ET-1 protein might be stored in secretory granules in neural cells as ET-like immunoreactivity co-localized with vasopressin and oxytocin-containing neurosecretory granules in rat posterior pituitary [57]. Our data show that human astrocytes exposed to the hypoxia (48–72 h) reacted very sensitively with an increase of ET-1 release. This could hint either at the rapid release of ET-1 stored in secretory vesicles or at *de novo* synthesis of ET-1.

Exposure of cultures to 24 h hypoxia did not significantly affect the ET-1 protein release from astrocytes. The vulnerability of astrocytes, assessed by apoptosis and LDH release, was previously shown to be increased from the time point 48 h whereas 24 h hypoxia did not affect the viability and apoptosis of astrocytes [52]. Application of EPO under normoxic conditions did not change ET-1 release from astrocytes while a significant decrease in ET-1 release could be observed during hypoxia in EPO-treated culture. Given that the effect of EPO on ET-1 release is mediated by EPOR, we hypothesize that the specific efficacy of EPO on ET-1 release only during hypoxia could be ascribed to the very low expression of EPOR during normoxic conditions. This hypothesis is supported by published data showing that only after acute and hypoxic brain injury a strong EPO and EPOR immunoreactivity appears in astrocytes, whereas in normal brain tissue only very weak expression of EPO/EPOR was detected in neurons but not in astrocytes [26].

Long-term hypoxia prominently affected survival of astrocytes, and ET-1 protein release from astrocytes significantly increased during 48–72 h hypoxia. This indicates that hypoxia-induced cell death may potentiate the release of ET-1 protein from astrocytes, which in turn could maintain the extent of the cellular damage upon hypoxia. The different ET-1 protein concentrations at time points 24, 48, and 72 h hint at the possible role of the cellular vulnerability to hypoxia. The maximum peak of ET-1 protein release was detected after 48 h incubation in hypoxia. This might indicate that the physiological ET-1 protein release of astrocytes in response to hypoxia occurs during the first 48 h, whereas later (>48 h) compensatory protective mechanisms may be activated to reduce the release of ET-1 protein and, hereby, to minimize the extent of the cellular damage under protracted hypoxic conditions.

The observed increase of EPO expression in rat astroglial cultures treated with ETA receptor antagonist BQ123 raised the question whether EPOR expression could be regulated by the ET system in CNS cells. Analysis of short-term (DIV7) astroglial cultures showed an increase in intensity of EPOR expression in astrocytes treated with BQ123 under normoxic conditions, and BQ123 treated cultures contained the round small cells positive only for EPOR but not for GFAP hinting at their non-glial origin. These cells may represent *bona fide* neural precursors that did not enter yet the glial lineage or bona fide neuronal precursors. As shown previously, astroglial primary cultures (APC) at day seven are composed of 2% tubulin beta III positive neurons, 1% ECs and 89% astrocytes under normoxic conditions. Upon hypoxia the total number of cells was reduced to 33% of the normoxic control, from which 21% ± 6% were ECs, 3% ± 0.93% neurons and 78% ± 15% astrocytes. [52]. It is reasonable to assume that the percentage of EPOR-positive neurons and neural precursors can increase upon BQ123 treatment. This suggestion is supported by the observation that BQ123 can increase the population of GFAP-negative neural precursors positive for nestin [30], a marker of astrocytes and undifferentiated neural precursor cells [58].

Hypoxia promoted the expression of EPOR in short term (DIV7) and long term (culture-aged [DIV21]) astroglial cultures. BQ123 did not potentiate the hypoxia-induced expression of EPOR in DIV21 astroglial cultures, while in DIV7 cultures EPOR expression was additionally increased in astrocytes by ETA blockade with BQ123. In view of the fact that the viability and EPO release of DIV7 astrocytes were only slightly affected by 24 h hypoxia, whereas DIV21 cells responded to hypoxia with a dramatic decrease in cell survival and increase in EPO release [59], it can be suggested that the maximum effect on EPOR expression in culture-aged cells might have been triggered by hypoxia alone and could not be potentiated by additional ETA blockade.

The effect of BQ123 on EPOR in DIV21 astroglia can probably be achieved only by increase in a number of newly generated cells with a strong capacity to express EPO and EPOR. This suggestion is concordant with the data showing an increase of round undifferentiated and neuron-like EPOR positive cells upon hypoxia and application of BQ123 in DIV21 cultures hinting at the pro-proliferative capacity of BQ123 under hypoxic conditions. Moreover, BQ123 applied to DIV21 astroglia cultures under hypoxia increased the differentiation of precursor cells as reflected in appearance of neuron-like process bearing cells.

## 4. Experimental Section

### 4.1. Rat Astroglial Primary Culture

Astroglia-rich primary cultures where prepared from the brains of newborn Wistar rats. At day 7 this culture contained 70%–85% GFAP-positive astroglial cells. For immunocytochemical studies, viable cells (1 × 10^6^) were seeded in culture dishes (60 mm in diameter) containing glass cover slips, whereas, for the proliferation/viability assay and measurement of caspase-3/−7 activity the cells were grown in 96-well microplates (10,000 cells/well). The cells were cultured in 90% Dulbecco’s modified Eagle’s medium containing 10% fetal calf serum (FCS), 20 μg/mL streptomycin sulphate and 20 units/mL penicillin G until day 5 *in vitro* (DIV) or DIV 19, the cells were cultured at 37 °C in an incubator containing air/10% CO_2_ atmosphere. At DIV 5 or DIV 19 the medium was removed and fresh medium containing different supplements was added. For immunocytochemical analyses the supplements were added at DIV 5 and DIV 19 without refreshing the cell culture medium. The samples were assayed as described below. The viability of astroglial cells was assessed by counts of viable cells under trypan blue (Sigma, Deisenhofen, Germany) staining in a Neubauer chamber.

### 4.2. Human Astrocytes (SV-40 FHAS) Culture

The immortalized human fetal astrocyte cell line (SV-FHAS) was kindly provided by Michael Weller (Department of Neurology, University Hospital Zurich, Zürich, Switzerland). For quantification of ET-1 in the cell culture supernatant 1 × 10^6^ cells were seeded in Petri dishes and cultured in Dulbecco’s Modified Eagle’s Medium (DMEM) containing 10% FCS, 20 μg/mL streptomycin sulphate and 20 units/mL penicillin G. Cultures were maintained under normoxic conditions in a humidified 5% CO_2_-atmosphere at 37 °C for 24 h to allow adherence. Thereafter, the medium was replaced by DMEM containing EPO (Neorecormon; 5 U/mL cell culture medium, Hoffmann-La Roche, Grenzach-Wyhlen, Germany) or BQ123 (Calbiochem, San Diego, CA, USA) or without supplements. The choice of 5 U/mL concentration of EPO was based on *in vivo* concentration of EPO in cerebrospinal fluid (5.148 U/mL) of stroke patients after intravenous administration of 33,000 IU (international units) of human recombinant EPO resulting in significant improvement of clinical outcome and neurological recovery [60]. This concentration was also shown to protect short and long term rat astroglial cultures against glutamate cytotoxicity and to increase astroglial clearance and metabolism of glutamate upon normoxic and hypoxic culture conditions [59]. The cells were immediately exposed to hypoxia (1% O_2_, 5% CO_2_, 94% N_2_) or incubated under normoxic conditions for 24, 48, or 72 h to be analyzed as described below.

### 4.3. Hypoxia and Stimulation with BQ123 and EPO

To investigate the influence of selective ETA/B-R-blockade and the effect of EPO on the alterations in proliferation, survival and apoptosis of astroglial cells caused by hypoxia, 5-day and 19-day-old rat astroglial primary cultures were treated for 48 h with BQ123, under normoxic and hypoxic culture conditions. Hypoxia treatment was introduced using an incubator containing 1% O_2_, 5% CO_2_, 94% N_2_, and maintained for 48 h at 37° C. BQ123 was dissolved in phosphate buffered saline (PBS).

### 4.4. Immunocytochemical Analysis

For the immunocytochemical studies 7- and 21-day-old astroglial primary cultures grown in Petri dishes (60 mm in diameter) containing coverslips under normoxic culture conditions and hypoxic conditions were treated with EPO or BQ123 48 h prior to the fixation. After fixation with −20 °C cold methanol, the cells were rinsed with PBS and incubated with mouse monoclonal anti-GFAP (diluted 1:10, Progen, Heidelberg, Germany) and rabbit anti EPO receptor (dilution 1:50, Santa Cruz, CA, USA) for 1 h at room temperature (RT). Subsequently, the cells were washed twice with PBS and labeled with the secondary antibodies: fluorescein isothiocyanate (FITC)-conjugated mouse anti-rabbit IgG diluted 1:100 and Cy3-conjugated goat anti-mouse IgG (1:800; Dianova Jackson Immunoresearch, West Grove, PA, USA) and incubated for 1 h at RT. The cells were washed with PBS containing 0.1% Triton X-100 (Sigma, Deisenhofen, Germany) and coated with Vectashield mounting medium (Vector Laboratories Burlingame, Burlingame, CA, USA) containing 4′.6 diamidino-2-phenylindole (DAPI). As for negative controls, samples were treated with secondary antibodies only. Microscopy was performed using Olympus fluorescence microscope BX 51 (Olympus Optical Co. Europe, Hamburg, Germany). Imaging was processed by digital camera F-View II and software AnalysisDOKU^®^ (Soft Imaging System GmbH, Leinfelden-Echterdingen, Germany).

### 4.5. Real Time RT-PCR

Total RNA from rat astroglial primary culture was isolated using RNeasy MiniKit (Qiagen, Hilden, Germany). The cDNA synthesis was performed using oligo dT15 and random hexamers as primers and avian myeloblastosis virus reverse transcriptase (PeqLab, Erlangen, Germany). After the washing procedure the RNS was quantified. PCR was carried out on the Lightcycler instrument with the FastStart DNA Master SYBR Green I kit (Roche, Mannheim, Germany). The PCR reaction mixture contained 1 μM sense and 1 μM antisense oligonucleotides with SYBR green I (Molecular Probes). The following primer sets were used: EPO (RefSeq Acc. No. NM_017001)–sense 5′-1272CACGAAGCCATGAAGACAGA1291-3′, antisense 5′-1372GGCTGTTGCCAGTGGTATTT1353-3′; *Peptidyl prolyl isomerase A* (*PPIA*, *cyclophilin A*, NM_017101)–sense 5′-166GGGGAGAAAG GATTTGGCTA185-3′, antisense 5′-422ACATGCTTGC CATCCAGCC404-3′. *PPIA* was used as a housekeeping gene. The relative expression ratio of EPO mRNA is presented as percentage of the mean of normoxic control samples.

### 4.6. Determination of ET-1 Protein

Human astrocytes were cultured as described above. The complete volume of 10 mL supernatant was collected from each plate, centrifuged to remove particulates and immediately frozen at −80 °C.

For quantitative determination of ET-1 protein a commercial available Elisa-Kit was used (QuantiGlo, human ET-1 Immunoassay, R&D, Wiesbaden, Germany).

To increase the detection limit, the supernatants were lyophilized and resolved in 1 mL of distilled water. To compensate the matrix effect appropriate standards concentrations of ET-1 protein and a blank probe were prepared in 10 mL of supplemented DMEM and analyzed like the samples. The test was performed according to the manufacturer’s manual. Results were obtained using a standard curve generated by a cubic-spline curve fit and were expressed as pg ET-1/10^6^ cells.

### 4.7. Statistical Analyses

The data obtained from three independent experiments for each experimental set were normalized to the mean of corresponding controls and evaluated in percentage of decrease or increase compared to control. Data are presented as mean ± SEM (*n* ≥ 5). One-way-ANOVA was used for each experiment. *p* < 0.05 was considered as significant (* *p* < 0.05, ** *p* < 0.01, *** *p* < 0.001). The statistical analyses were performed on normalized data. To compare the treated samples with controls and among each other Dunnett’s Multiple Comparison Test was used.

## 5. Conclusions

Summarizing the data of the present study we conclude that the interactions of the local ET and EPO system are characterized by: (1) Influence of EPO on the release and possibly the synthesis of ET-1 mediated via EPOR which in turn is up-regulated by hypoxia; (2) The increase of *EPO* mRNA expression by hypoxia which is potentiated by the selective ETA receptor blockade through BQ123 in DIV21 astroglial culture; and (3) Induction of EPOR expression in precursor cells and DIV7 astrocytes by ETA receptor blockade.

The similarities of the protective effects mediated by EPO and ETA receptor blockade and the fact that the combined use of these substances (EPO and BQ123) can additionally improve the survival of astroglial cells and the generation of neurons as well as neural precursors guide to the suggestion that combined administration of ETA antagonists plus EPO may provide a strong protection of CNS cells during hypoxia associated tissue damage.
